# Rapid Assessment of Functional Avidity of Tumor-Specific T Cell Receptors Using an Antigen-Presenting Tumor Cell Line Electroporated with Full-Length Tumor Antigen mRNA

**DOI:** 10.3390/cancers12020256

**Published:** 2020-01-21

**Authors:** Diana Campillo-Davo, Maarten Versteven, Gils Roex, Hans De Reu, Sanne van der Heijden, Sébastien Anguille, Zwi N. Berneman, Viggo F. I. Van Tendeloo, Eva Lion

**Affiliations:** 1 Tumor Immunology Group, Laboratory of Experimental Hematology, Vaccine & Infectious Disease Institute (VAXINFECTIO), Faculty of Medicine and Health Sciences, University of Antwerp, 2610 Wilrijk, Antwerp, Belgium; 2 Division of Hematology, Antwerp University Hospital, 2650 Edegem, Antwerp, Belgium; 3 Center for Cell Therapy & Regenerative Medicine, Antwerp University Hospital, 2650 Edegem, Antwerp, Belgium

**Keywords:** model antigen-presenting cell, T-cell functional avidity, full-length antigen mRNA electroporation, Wilms’ tumor 1, cancer immunotherapy

## Abstract

The functional avidity of T-cell receptor (TCR)-engineered T cells towards their cognate epitope plays a crucial role in successfully targeting and killing tumor cells expressing the tumor-associated antigen (TAA). When evaluating in vitro functional T-cell avidity, an important aspect that is often neglected is the antigen-presenting cell (APC) used in the assay. Cell-based models for antigen-presentation, such as tumor cell lines, represent a valid alternative to autologous APCs due to their availability, off-the-shelf capabilities, and the broad range of possibilities for modification via DNA or messenger RNA (mRNA) transfection. To find a valuable model APC for in vitro validation of TAA Wilms’ tumor 1 (WT1)-specific TCRs, we tested four different WT1 peptide-pulsed HLA-A2+ tumor cell lines commonly used in T-cell stimulation assays. We found the multiple myeloma cell line U266 to be a suitable model APC to evaluate differences in mean functional avidity (EC50) values of transgenic TCRs following transfection in 2D3 Jurkat T cells. Next, to assess the dose-dependent antigen-specific responsiveness of WT1 TCR-engineered 2D3 T cells to endogenously processed epitopes, we electroporated U266 cells with different amounts of full-length antigen *WT1* mRNA. Finally, we analyzed the functional avidity of WT1 TCR-transfected primary CD8 T cells towards *WT1* mRNA-electroporated U266 cells. In this study, we demonstrate that both the APC and the antigen loading method (peptide pulsing versus full-length mRNA transfection) to analyze T-cell functional avidity have a significant impact on the EC50 values of a given TCR. For rapid assessment of the functional avidity of a cloned TCR towards its endogenously processed MHC I-restricted epitope, we showcase that the TAA mRNA-transfected U266 cell line is a suitable and versatile model APC.

## 1. Introduction

T-cell receptor (TCR) gene therapy is a promising strategy in cancer immunotherapy, capitalizing on the use of TCR-engineered T cells targeting tumor-associated antigens (TAAs) expressed by cancer cells [1]. An essential element for the success of this type of therapy is the ability of TCR-engineered T cells to recognize the TAA, even at low epitope densities [2,3]. The threshold of activation of a T cell, defined as functional avidity, is a measurement of its effector response towards a particular surface density of the epitope [4]. Usually, it is evaluated in vitro by analyzing the response of T cells in peptide titration experiments. In this type of experiments, target cells are pulsed with decreasing concentrations of major histocompatibility complex (MHC)-binding peptides. The mean functional avidity, usually described by EC50, represents the peptide dose at which half-maximal activation of the T-cell population is reached. This value depends on the affinity and avidity of the TCR for its cognate peptide-MHC (pMHC) ligand and, therefore, it varies between different T-cell clones or TCR-engineered T cells. Generally, higher functional avidities—i.e., lower EC50 values—are linked to the recognition of lower epitope densities on the surface of antigen-presenting cells (APCs), and, thus, to better responses towards those cells [5,6]. Hence, the analysis of antigen-specific T-cell responses is vital at a clinical and research level to obtain the best TCRs for adoptive T-cell therapies [7,8].

Measurement of T-cell functional avidity, however, can be challenging due to the vast array of analytical methods and the use of different types of cells presenting the antigen. Assays for the measurement of in vitro antigen-specific T-cell functional activity include direct cytotoxicity analysis by chromium (^51^Cr) release [9] or flow cytometry-based killing assays [10], detection of intracellular expression of cytokines such as interferon-gamma (IFN-γ) or interleukin-2 (IL-2) [11,12], IFN-γ or granzyme B enzyme-linked immunospot (ELISpot) assays [13] and enzyme-linked immunosorbent (ELISA) assays [14], mobilization of CD107a [15,16], and upregulation of activation markers, e.g., CD69 or CD137 [17]. In some models using TCR-deficient Jurkat cells, TCR activation is measured by the TCR-triggered expression of the green fluorescent protein (GFP) [18] or a combination of fluorescent proteins for the analysis of different transcription factors associated with TCR signaling [19]. Despite the multiple ways to analyze T-cell functional avidity, little is known about the impact that an APC may have on the result. This is important because T-cell activity may vary depending on the epitope density displayed by the APC, but also on the ability of an APC to promote T-cell activation. Among the multiple possibilities, cells of autologous origin, such as peripheral blood mononuclear cells (PBMCs), monocyte-derived dendritic cells (DCs) and B-lymphoblastoid cell lines represent the most common APCs in T-cell activation assays. Non-autologous cell-based models of APCs, including tumor cell lines such as T2 or K562 cells, are an alternative to the costly and laborious production of autologous APCs [20]. They represent an off-the-shelf approach that can be easily maintained, readily available, and modified as per request. Moreover, model APCs can be engineered with plasmid vectors or messenger RNAs (mRNAs) that encode the tumor antigens of interest. In particular, electroporation of antigen-encoding mRNA is a rapid and efficient method to induce neo-expression of the antigen in APCs. This technique allows the induction of multi-epitope T-cell responses, for example, in cancer patients following therapeutic vaccination with antigen-loaded DCs, such as the Wilms’ tumor 1 (WT1) protein [7,21]. WT1 is a transcription factor overexpressed in leukemia and many solid tumors, but also present in healthy tissues [22]. Unfortunately, as an auto-antigen, T cells targeting self-TAAs such as WT1 with high avidity are scarce due to the negative selection that occurs in the thymus [23].

In the context of WT1-targeted adoptive TCR-engineered T-cell immunotherapies, methods to correctly asses the functional avidity of T cells engineered with WT1-specific TCRs are crucial for their success. In this study, we aimed to develop a reliable APC model for the evaluation of endogenously processed WT1 peptides and the avidity of WT1-specific TCRs. We analyzed the use of the HLA*A2:01-positive multiple myeloma cell line U266 as a tool for the rapid assessment of HLA-A2-restricted WT1-specific T-cell responses following electroporation with full-length *WT1* mRNA, in comparison with WT1 peptide loading. To the best of our knowledge, this is the first study comparing exogenous peptide-loading and full-length antigen mRNA electroporation of target cells to study the functional avidity of epitope-specific TCR-redirected T cells.

## 2. Results

### 2.1. Quantitation of WT1-Presenting Potential Odel APC

**Figure 1:**
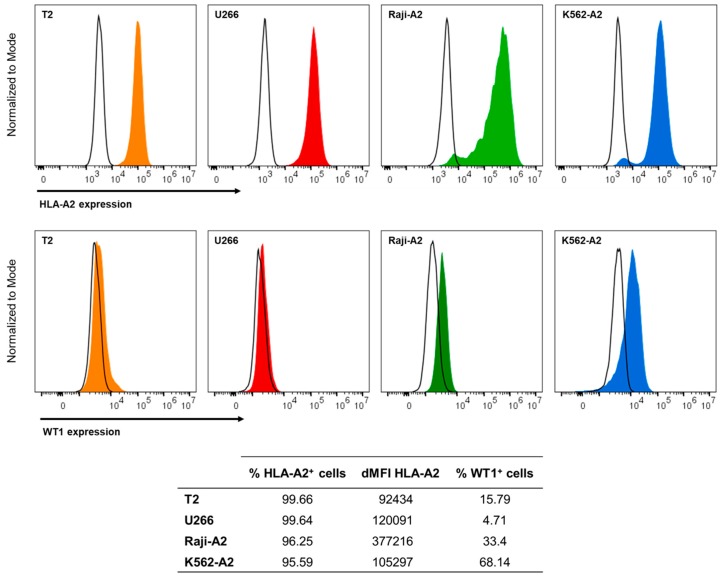
HLA-A2 and WT1 expression on four model antigen-presenting cell (APC) lines. Histograms (relative to mode) show the surface expression of HLA-A2 (upper panel) and the intracellular expression of WT1 (lower panel) of T2 (orange), U266 (red), Raji-A2 (green), and K562-A2 (blue) cell lines. HLA-A2 or WT1 expression (filled histograms) and isotype control (black line). The table shows HLA-A2 delta median fluorescence intensity (dMFI) values and percentage of HLA-A2 positive cells minus isotype staining (upper histograms) or percentages of WT1 positive cells minus isotype staining (lower histograms) for each cell line. HLA-A2, human leukocyte antigen A*02:01; WT1, Wilms’ tumor 1 protein.

To evaluate the capacity of different cell lines to be used as model APCs for presentation of WT1-derived epitopes by HLA-A2, the expression of surface HLA-A2 and natural intracellular WT1 proteins of four potential cell lines was quantified: T2 [24], U266 [25], K562-A2 [26] and Raji-A2 [27] cells (Figure 1). All cell lines expressed HLA-A2, with percentages ranging from 95% to 99% of HLA-A2-positive cells (Figure 1, upper panel). With regards to the number of HLA-A2 molecules per cell, denoted as delta median fluorescence intensity (dMFI), T2 cells expressed the lowest levels of HLA-A2 molecules. On the contrary, Raji-A2 showed the highest levels of expression, whereas U266 and K562-A2 cells showed similar intermediate levels. Confirming literature, K562-A2 was the only cell line that clearly expressed WT1 (68.14% WT1^+^), whereas T2 and Raji-A2 cells expressed moderate amounts of the antigen (15.79% and 33.4% WT1^+^, respectively) and U266 cells the lowest amounts (4.71% WT1^+^) (Figure 1, lower panel).

### 2.2. Functional Avidity of WT1-Specific T Cells Drastically Differs Depending on the APC Used

**Figure 2:**
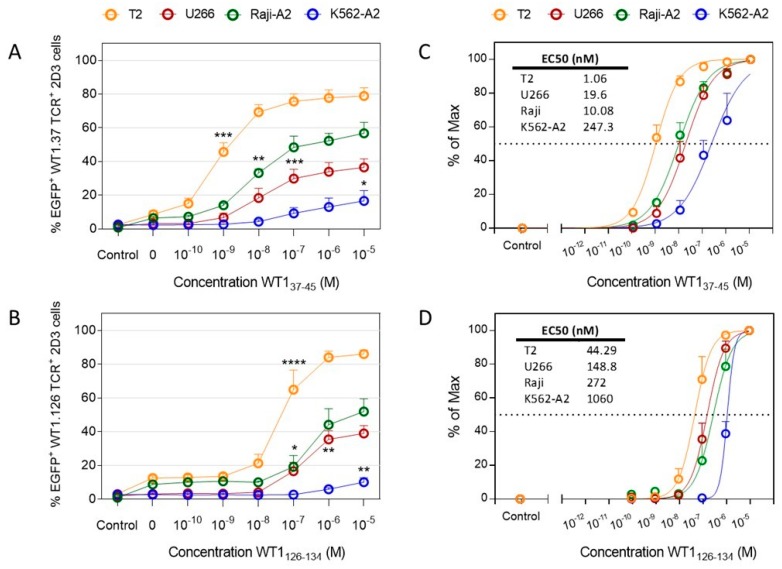
Epitope-specific T-cell activation by four model APC lines. Epitope-specific TCR activation was measured by expression of EGFP after WT1_37–45_ (**A**,**C**) or WT1_126–134_ (**B**,**D**) peptide-specific TCR-transfected 2D3 cells were cultured for 18–22 h with model APCs T2, U266, Raji-A2 or K562-A2 cells that were pulsed with decreasing concentrations of WT1 peptide. Control depicts unstimulated 2D3 cells only. Graphs show the results of three to five independent replicates, showing (**A**,**B**) mean % (± SEM) of EGFP positive cells and (**C**,**D**) % of maximal EGFP expression (± SEM). (**A**,**B**) Data were analyzed using one-way ANOVA followed by Dunnett’s post hoc test (comparing to non-peptide pulsed cells). EC50, the concentration of WT1 peptide at which 50% of the maximal EGFP expression is reached. *, *p* < 0.05; **, *p* < 0.01; ***, *p* < 0.001; and ****, *p* < 0.0001.

To analyze the WT1 peptide-presenting capacity of the four model APC candidates, we used an in-house developed T-cell model assay, based on TCR-deficient CD8^+^ Jurkat 2D3 cells that are electroporated with TCRαβ-encoding mRNAs and express enhanced green fluorescent protein (EGFP) via nuclear factor of activated T cells (NFAT) upon antigen-specific TCR triggering [28,29]. Transgenic TCR expression for two HLA-A2-restricted TCRs directed against two epitopes of the WT1 protein, WT1_37−45_ and WT1_126−134_ (WT1.37 and WT1.126 TCR, respectively), was maximal for both TCRs 24 h after electroporation (92.75 ± 1.5% WT1.37 TCR^+^ and 94.48 ± 0.67% WT1.126 TCR^+^ 2D3 cells; Supplementary Figure S1A). Pulsed with decreasing concentrations of WT1_37–45_ or WT1_126–134_ peptides, the four model APCs were cultured with their respective *WT1 TCR* mRNA-electroporated 2D3 cells (Figure 2). The peak values of EGFP expression in 2D3 cells, corresponding to maximal T-cell activation, were detected with the highest peptide concentration for all cell lines (Figure 2A,B). The intensity of the T-cell response differed for both WT1-specific TCRs and depended on the APC type. When cultured with peptide-pulsed T2 cells, the highest percentages of EGFP^+^ 2D3 cells were reached as compared to U266 cells, Raji-A2, and K562-A2 cells, the latter promoting the poorest T-cell activation against both WT1 peptides. T2 cells, together with Raji-A2, displayed higher background levels of non-specific activation for both WT1.37 and WT1.126 TCR-electroporated 2D3 cells. Compared to the response observed with non-pulsed model APCs, the threshold of activation with T2 cells was reached at 10^−9^ M for WT1.37 peptide (*p* = 0.0002; Figure 2A) and 10^−7^ M for WT1.126 (*p* = 0.0001; Figure 2B). In the case of U266, significant differences were detected at 10^−7^ M for both peptides (*p* = 0.0007 and 0.0456, respectively). As for Raji-A2 cells, the threshold of activation was reached at 10^−8^ M for WT1.37 peptide (*p* = 0.0017) and 10^−6^ M for WT1.126 (*p* = 0.0015). WT1.37 and WT1.126 TCR^+^ 2D3 cells were only able to significantly respond to K562-A2 cells pulsed with a concentration of 10^−5^ M for both WT1 peptides (*p* = 0.0284, and *p* = 0.0012, respectively). Uniformly comparing all cell lines, percentages of EGFP expression were normalized for the calculation of EC50 values (Figure 2C,D). In the same line, the EC50 values strongly varied between cell lines. Again, T2 cells were capable of promoting the best T-cell response for both WT1-specific TCRs (EC50: 1.06 nM for WT1.37 TCR and 44.29 nM for WT1.126 TCR). On the opposite side, peptide-pulsed K562-A2 cells induced T-cell responses at higher concentrations (EC50: 247.3 nM for WT1.37 TCR and 1060 nM for WT1.126 TCR). In the middle range, U266 and Raji-A2 cells promoted half-maximal responses at similar concentrations for the WT1.37 TCR (EC50 U266: 19.6 nM; EC50 Raji-A2: 10.08 nM), and WT1.126 TCR (EC50 U266: 148.8 nM; EC50 Raji-A2: 272 nM). These results show that for the same T-cell population expressing an antigen-specific TCR, the APC chosen for the peptide titration experiments has a pivotal role in the thresholds of T-cell activation.

### 2.3. Mimicking Endogenous WT1 Expression

**Figure 3:**
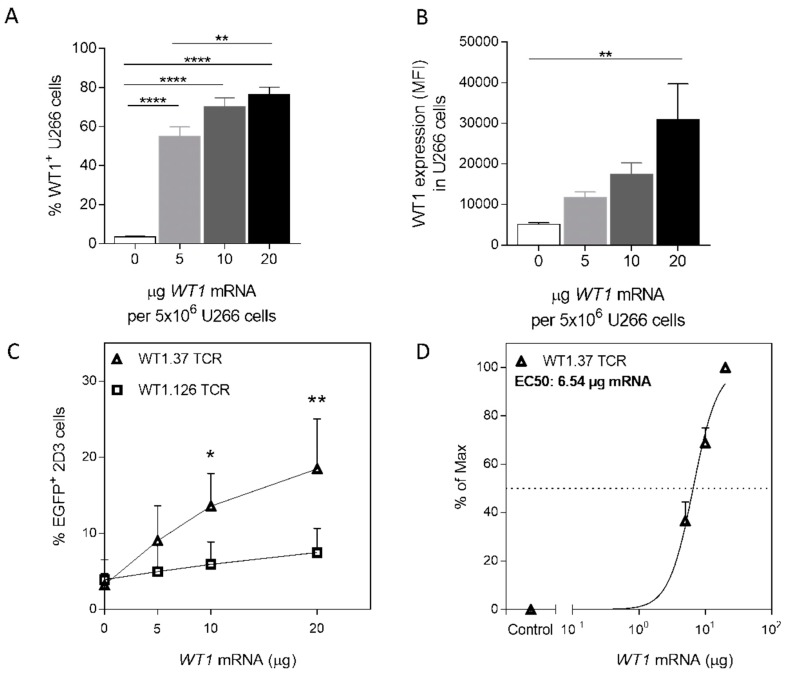
Epitope-specific TCR-engineered 2D3 cells can recognize full-length antigen *WT1* mRNA-electroporated U266 cells in a dose-dependent manner. (**A**,**B**) Intracellular expression of WT1 is shown for U266 cells 24 h after electroporation with increasing amounts of *WT1* mRNA per 5 × 10^6^ U266 cells. (**C**,**D**) 2D3 cells were electroporated with WT1_37–45_- or WT1_126–134_-specific *TCR* mRNAs. Specific activation was detected by NFAT-promoted EGFP expression in 2D3 cells after 18–22 h co-culture with U266 cells electroporated with increasing amounts of *WT1* mRNA. Graphs show the mean percentage of WT1^+^ U266 cells ± SEM (**A**), the median fluorescence intensity (MFI) of U266 for WT1 expression ± SEM (**B**), the percentage of maximal EGFP expression ± SEM (**C**) and EC50, the amount of *WT1* mRNA at which 50% of the maximal EGFP expression is reached (**D**) of 3–4 independent replicates. (**A**,**B**) One-way ANOVA followed by Tukey’s post hoc test. (**C**) One-way ANOVA followed by Dunnett’s post hoc test (comparing to mock-electroporated cells). *, *p* < 0.05; **, *p* < 0.01; and ****, *p* < 0.0001.

Developing a model that can mimic the endogenous processing of WT1 in tumor cells in a controlled manner, among the four cell lines analyzed, T2 and K562-A2 cells are not model candidates because the former are unable to present internally-processed peptides and the latter are intrinsically highly positive for WT1. U266 and Raji-A2 cells generated similar EC50 values; however, they differed in natural WT1 expression levels. We selected the U266 cell line as the candidate model for further analysis due to the lower percentage of WT1 positive cells. Therefore, U266 cells, which naturally express HLA-A2 and minimal levels of WT1, were electroporated with increasing amounts of *WT1* mRNA as the best model for presentation of internally-processed WT1 peptides (Figure 3). The increment in mRNA load resulted in an increase in the percentage of cells expressing the protein (Figure 3A), reaching the highest value of WT1^+^ U266 cells (76.5 ± 3.66%) upon electroporation of 20 µg of *WT1* mRNA. A significant difference between the 5 µg and 20 µg mRNA condition in % of WT1-expressing cells was observed (*p* = 0.0078), demonstrating a dose-response dependency. Likewise, WT1 protein expression per cell increased with increasing mRNA concentrations after electroporation (Figure 3B). Next, we assessed the antigen-presenting capacity of the *WT1* mRNA-electroporated U266 cells in combination with *WT1 TCR* mRNA-electroporated 2D3 cells. EGFP expression by WT1.37 TCR^+^ 2D3 cells (Figure 3C, triangles) was significantly higher than mock electroporation (0 µg *WT1* mRNA) when using 10 µg (13.56 ± 2.15%; *p* = 0.0348) and 20 µg (18.48 ± 3.28%; *p*= 0.0025) *WT1* mRNA, but not with 5 µg (9.04 ± 2.28%; *p* = 0.3245). This indicates that WT1.37 epitope density on U266 cells after electroporation with 5 µg of *WT1* mRNA/5 × 10^6^ cells per electroporation is not enough to surpass the threshold for WT1.37 TCR activation. On the other hand, WT1.126 TCR^+^ 2D3 cells (Figure 3C, circles) were not able to respond significantly to any of the amounts of *WT1* mRNA used. Analyzing the amount of mRNA at which 50% of maximal EGFP response was obtained, WT1.37 TCR-engineered T cells showed an EC50 value at 6.54 µg *WT1* mRNA-electroporated U266 (Figure 3D). This information could support indicating the minimum dosage of mRNA that should be used in APCs for the evaluation of specific T-cell clones or TCR-engineered T cells.

### 2.4. WT1 mRNA-Electroporated U266 Cells Activate WT1-Specific TCR-Redirected Primary Human CD8 T Cells in a Dose-Dependent Manner

**Figure 4:**
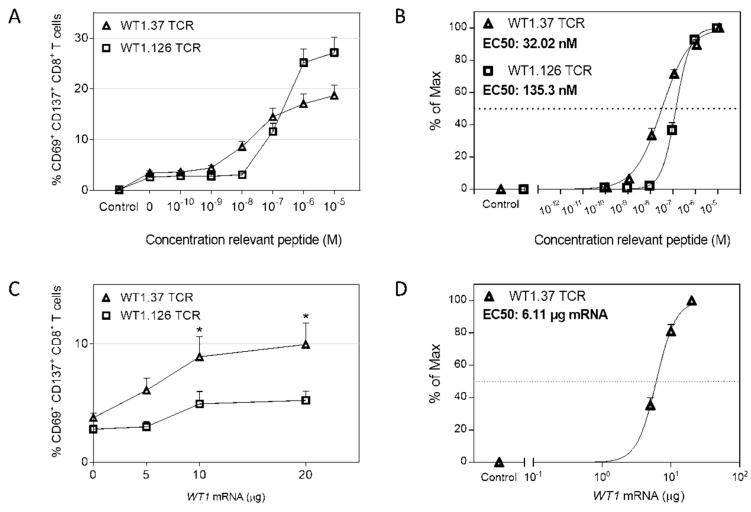
Analysis of functional avidity of WT1 epitope-specific primary CD8 T cells using WT1 peptide-pulsed and *WT1* mRNA-electroporated U266 cells. Surface expression of both CD69 and CD137 activation markers was measured on WT1.37 (triangles) and WT1.126 (squares) peptide-specific DSE-engineered primary CD8 T cells 24 h after co-culture with U266 cells that were either pulsed with decreasing (**A**,**B**) concentrations of WT1_37–45_ or WT1_126–134_ peptide, or *WT1* mRNA-electroporated (**C**,**D**). T cells only condition was used as a control. Graphs show mean ± SEM of % CD69/CD137 double positive CD8 T cells (**A**,**C**) or % of maximal CD69/CD137 expression ± SEM (**B**,**D**) for 6–8 donors. EC50, the concentration of WT1 peptides or amounts of electroporated *WT1* mRNA at which 50% of the maximal upregulation of CD69 and CD137 activation markers is reached. (**A**,**C**) One-way ANOVA followed by Dunnett’s post hoc test (comparing to non-peptide pulsed or mock-electroporated cells). *, *p* < 0.05.

Further evaluating the antigen-presenting capacity of U266 cells, the functional avidity of unstimulated primary human CD8 T cells was analyzed in the context of WT1.37 and WT1.126 peptides using our in-house developed double sequential electroporation (DSE) T cell assay [28]. In brief, purified CD8 T cells were subjected to DsiRNA-*TCR* mRNA to downregulate the expression of endogenous TCR, before codon-optimized WT1-specific *TCR* mRNA electroporation. For both WT1.37 and WT1.126 TCRs, high TCR expression was achieved 24 h after *TCR* mRNA electroporation (66.9 ± 5.345% WT1.37/HLA-A2 tetramer^+^ and 72.4 ± 3.88% WT1.126/HLA-A2 tetramer^+^ for eight donors; Supplementary Figure S1B). These WT1 TCR-engineered CD8 T cells were co-cultured with peptide-pulsed or *WT1* mRNA-electroporated U266 and analyzed for WT1-specific CD8 T-cell activation and functional avidity by upregulation of CD69 and CD137 activation markers (Figure 4). For the WT1.37 peptide (Figure 4A), significant differences compared to the non-peptide pulsed U266 cells were still detected at a peptide concentration of 10^−8^ M (8.61 ± 1.07% CD69/CD137^+^; *p* = 0.0313), whereas the signal was lost at 10^−9^ M (4.4 ± 0.44% CD69/CD137^+^; *p* = 0.9931). In analogy with 2D3 cells, primary CD8 T cells electroporated with WT1.126 TCR were less sensitive to lower concentrations of the cognate peptide, compared to WT1.37 TCR^+^ CD8 T cells. U266 cells pulsed with a WT1.126 peptide concentration of minimal 10^−7^ M elicited significant primary T-cell activation (11.59 ± 1.64% CD69/CD137^+^; *p* = 0.0010). EC50 values of functional avidity for WT1.37 (32.02 nM) and WT1.126 (135.3 nM) TCR-engineered primary CD8 T cells (Figure 4B) were comparable to those obtained for 2D3 cells (19.6 nM and 148.8 nM, respectively). These findings confirm the usefulness of U266 cells in peptide-pulsing assays for the assessment of the functional avidity of primary TCR-redirected T cells. With regard to the *WT1* mRNA-electroporated U266 cells (Figure 4C), only WT1.37 TCR-engineered primary CD8 T cells significantly responded to 10 µg (8.92 ± 1.71% CD69/CD137^+^; *p* = 0.0383) and 20 µg of *WT1* mRNA (9.96 ± 1.82% CD69/CD137^+^; *p* = 0.0119). No significant differences with WT1.126 TCR-engineered CD8 T cells towards U266 cells electroporated with increasing amounts of electroporated *WT1* mRNA were observed. In the case of WT1.37 TCR^+^ CD8 T cells, 6.11 µg of *WT1* mRNA would be needed to reach upregulation of CD69 and CD137 in half of the maximal percentage of cells (Figure 4D), which is in line with the results using the 2D3 cell line (6.54 µg for WT1.37 TCR^+^; Figure 3D). Taken together, these findings show that evaluation of T-cell functional avidity with WT1 peptide-pulsed or *WT1* mRNA-electroporated U266 cells remains constant for the TCRs analyzed regardless of the source of T cells, and that this system can help to distinguish TCRs that will respond to epitope densities of naturally processed WT1 protein. Hence, the application of U266 cells as a suitable APC model for WT1 antigen-specific T-cell assays.

## 3. Discussion

Cell-based model APCs represent a valid alternative to autologous APCs and commonly used methods for analyzing antigen-specific T-cell activation status, for promoting ex-vivo T-cell expansion, and for the immunomonitoring of T-cell responses in the course of a viral infection or against cancer antigens in clinical trials [30]. To better understand the effect of the model APCs in the measurement of functional avidity of T cells, we compared four different model APC tumor cell lines (T2, U266, Raji-A2, and K562-A2). We showed a differential response in functional avidity of WT1-specific TCR-engineered T cells against different peptide-pulsed model APC tumor lines. This information is vital for an accurate calculation of T-cell responses when selecting T-cell clones or TCR-engineered T cells for cancer immunotherapy. Two of these cell lines, T2 and K562-A2, are routinely used in T-cell assays. In particular, T2 cells are widely used in peptide-MHC class I binding assays [31] due to their deficiency in transporter associated with antigen presentation (TAP). This complex is involved in the translocation of proteasome-processed peptides from the cytosol into the lumen of the ER [32]. The TAP deficiency in T2 cells results in MHC instability and reduction of nearly 70% of HLA-A2 surface expression [33] that would explain the lower dMFI for HLA-A2 in these cells. The absence of TAP proteins also prevents the internal loading of TAP-dependent peptides onto the MHC molecules, making the HLA-A2 proteins available for the addition of exogenous peptides. Since endogenously processed and exogenously added peptides in peptide pulsing assays compete for the HLA-A2 molecules available [34], it is not surprising that TAP-deficient T2 cells outperformed Raji-A2, U266 cells, and K562-A2. However, manifested by the very low threshold of functional avidity when using peptide-pulsed T2 cells, they may reflect a non-physiological model that does not represent the actual T-cell functionality. This fact could lead to an overestimation of the T-cell functional avidity and to the selection of T-cell clones or TCRs that are of lower avidity towards more natural peptide-presenting target cells, particularly when screening for high avidity T-cell clones able to recognize tumor cells endogenously expressing, processing and presenting relevant tumor antigens.

A comparison between T2, K562-A2 and autologous B-LCL cells in a flow cytometry-based assay of T-cell killing capacity, showed that T cells cultured with peptide-pulsed T2 cells elicited a better response than those cultured with K562-A2 or B-LCL [35]. These results also indicate that T2 cells present a supraphysiological epitope density after incubation with exogenously added peptides. Interestingly, K562-A2 cells failed to properly activate T cells in our system. As reported by Britten et al., this cell line is a suitable model for interferon (IFN)-γ ELISpot assays [26]. Britten and colleagues transduced K562-A2 cells with tyrosinase for its endogenous expression or exogenously pulsed them with tyrosinase-derived peptides. Therefore, T-cell responses to the natural expression of the ligand were not evaluated. Moreover, K562 cells naturally express WT1; thus, it does not represent a convenient model APC for the customization of *WT1* mRNA intracellular levels. In this regard, Raji-A2 cells also did express the WT1 protein, albeit at very low levels. This fact, together with the dramatic overexpression of HLA-A2, tips the balance in favor of WT1-negative, naturally HLA-A2 expressing U266 cells.

In our study, the discrepancy in T-cell responses observed with the different cell lines highlights the importance of the APC when assessing functional avidity, but also the influence of the source of the studied epitope. In many types of malignancies, tumor cells downregulate the expression of MHC proteins [36] or have deficiencies in their antigen processing pathways [37], which negatively impacts the presentation and density of peptides on their surface. Since the expression of a precise pMHC complex on the surface of the model APC depends on its capability to internally process full antigens, the sole addition of exogenous synthetic peptides for T-cell assays may provide an incomplete and potentially misleading scenario for the analysis of T-cell functional avidity. We show that U266 cells can be efficiently electroporated with full-length antigen *WT1* mRNA. The electroporation of higher amounts of mRNA was correlated with an increase in WT1 expression. This represents a flexible system in which different amounts of mRNA can be tested prior to clinical trials with full-length tumor antigen mRNA-electroporated DCs. Moreover, *WT1* mRNA-electroporated U266 cells could be a useful alternative cell-based antigen presentation model to DCs [38,39], K562 cells [39] or PBMCs [40] for the oligo-clonal detection of WT1-specific T cell populations and immunomonitoring of T-cell responses in full-antigen mRNA-electroporation DC vaccination trials. The generation of autologous APCs for T-cell assays is not always possible and often entails a lengthy process required for every donor. This could be overcome by the use of U266 cells as model APCs. Moreover, epitope-specific T cell responses induced by *WT1* mRNA-electroporated U266 cells can be compared to a peptide-titration curve using the same cell line. Another advantage of *WT1* mRNA-electroporated U266 is the possibility of off-the-shelf production by freezing the cells after electroporation.

Our study also confirms the suitability of 2D3 cells for the analysis of TCR avidity, thanks to their expression of human CD8 co-receptor, the absence of a native TCRαβ, the simplicity to engineer them with an antigen-specific TCR, and the expression of EGFP upon TCR triggering. The lack of endogenous TCR eliminates the possibility of TCR mispairing between endogenous and transgenic TCRs [41]. Therefore, EGFP expression can be directly correlated with the degree of introduced TCR triggering, i.e., the capacity of different APCs to present a peptide and to activate T cells. These findings are in accordance with previous reports showing that tetramers allow the quantification of antigen-specific T cells, but do not always provide accurate data on the functionality of T cells [42,43,44,45]. Regarding primary human CD8 T cells, activation markers enable the identification of all responder T cells after TCR triggering. One of the most common activation markers in flow cytometric analysis is CD137. Combined with CD69, CD137 is a powerful and sensitive tool to measure epitope-specific T cells regardless of the T-cell state of differentiation or subset [46].

## 4. Materials and Methods

### 4.1. Cell Lines and Primary Cells

The TCRαβ-deficient, CD8αβ and NFAT-EGFP stably-transfected T cell acute leukemia 2D3 cell line [28,29] was kindly provided by Prof. Haruo Sugiyama (Osaka University Graduate School of Medicine, Osaka, Japan) and maintained in Roswell Park Memorial Institute 1640 (RPMI) culture medium (Life Technologies, Merelbeke, Belgium) supplemented with 10% FBS. The HLA-A*02:01-positive WT1-negative human transporter associated with antigen presentation (TAP)-deficient lymphoblastoid T2 cell line was kindly provided by Dr. Pierre Van der Bruggen (Ludwig Institute for Cancer Research, Brussels, Belgium). U266 is an HLA-A*02:01-positive, WT1-negative multiple myeloma cell line and was a kind gift from Dr. Wilfred T.V. Germeraad (GROW School for Oncology & Developmental Biology, Maastricht University, Maastricht, The Netherlands). The HLA-A*02:01-transduced Burkitt’s lymphoma Raji-derived Raji-A2 cell line was kindly provided by Dr. Mirjam Heemskerk (Leiden University Medical Center, Leiden, The Netherlands). The HLA-A*02:01-transduced human chronic myelogenous leukemia K562-derived K562-A2 cell line was a kind gift from Dr. Cedrik Britten (R&D Oncology, GlaxoSmithKline, Stevenage, UK). T2, U266, Raji-A2, and K562-A2 cells were cultured in Iscove’s Modified Dulbecco’s Medium (IMDM; Life Technologies,) supplemented with 10% FBS. All cell lines were maintained in a logarithmic growth phase at 37 °C in a humidified atmosphere supplemented with 5% CO_2_.

Blood samples of healthy anonymous donors were purchased from the Blood Service of the Flemish Red Cross (Mechelen, Belgium) following the approval by the Ethics Committee of the Antwerp University Hospital and the University of Antwerp (reference number 16/35/357). Peripheral blood mononuclear cells were isolated by density gradient centrifugation using Ficoll-Paque PLUS (GE Healthcare, Diegem, Belgium), and CD8 T cells were selected using human CD8 magnetic microbeads for magnetic-activated cell sorting (MACS) according to the manufacturer’s instructions (Miltenyi Biotec, Leiden, The Netherlands). The purity of CD8 T cells after MACS isolation was analyzed using a CytoFLEX flow cytometer (Beckman Coulter, Suarlée, Belgium) after staining with FITC-labeled anti-CD8, PE-conjugated anti-CD4 and PerCP-conjugated anti-CD3 monoclonal antibodies (mAbs; Becton-Dickinson (BD) Biosciences, Erembodegem, Belgium). After MACS isolation, CD8 T cells were centrifuged and resuspended in cryopreservation medium consisting of fetal bovine serum (FBS; Life Technologies) supplemented with 10% dimethyl sulfoxide (DMSO; Sigma-Aldrich, Diegem, Belgium). Aliquots of 20–35 × 10^6^ cells/mL were transferred to Mr. Frosty freezing containers (Thermo Fisher Scientific, Erembodegem, Belgium) filled with isopropyl alcohol (Yvsolab, Turnhout, Belgium) and kept in a −80 °C freezer for at least seven days up to three weeks. Aliquots were thawed in pre-warmed AIM-V (Life Technologies) supplemented with 10% human AB serum (hAB; Life Technologies) and rested for at least one hour in a humidified 5% CO_2_ incubator at 37 °C.

### 4.2. In Vitro Transcription of mRNA

The cloning of WT1-specific *TCR* genes, generation of the pST1 DNA plasmids containing the TCR constructs and generation of WT1-specific *TCR* mRNA by in vitro transcription (IVT) were performed as previously described [28,29]. Clinical-grade codon-optimized Sig-DC-LAMP *WT1* mRNA encoding isoform D of WT1 [21] was purchased from eTheRNA immunotherapies (Niel, Belgium).

### 4.3. Electroporation

Electroporation of 2D3 cells with WT1-specific *TCR* mRNA was performed as previously described [28]. Double sequential electroporation (DSE) of human primary CD8 T cells was performed following [28], with minor modifications. Briefly, 10 or 20 × 10^6^ thawed viable human primary CD8 T cells were resuspended in 200 or 400 μL of serum-free Opti-MEM medium (Life Technologies) after thawing and transferred to a 4 mm-gap electroporation cuvette (Cell Projects, Harrietsham, UK). Next, cells were electroporated with 16 or 32 μL of a pool containing 100 μM of TRAC- and TRBC-specific DsiRNAs (Integrated DNA Technologies) in a ratio of 1:1. After electroporation, cells were transferred to pre-warmed AIM-V medium supplemented with 10% hAB, rested at 37 °C in a humidified atmosphere supplemented with 5% CO_2_ for at least 20 min, centrifuged (300× *g*, 3 min), transferred to 6-well plates and then incubated for 24 h. Second electroporation with in vitro transcribed mRNA was performed following the same protocol, using 1 μg of mRNA per 10^6^ cells. For the electroporation of U266 cells, 5 × 10^6^ viable cells were washed once with Opti-MEM I medium (Life Technologies), resuspended in 200 μL of the same medium, and then transferred to 4 mm-gap cuvettes (Cell Projects). Next, 5, 10 or 20 μg of clinical-grade IVT *WT1* mRNA was added to the cells before electroporation. Cells were electroporated in a Gene Pulser Xcell™ device (Bio-Rad Laboratories, Temse, Belgium) using the Time constant protocol (300 V, 8 ms, one pulse). After electroporation, all cells were transferred to pre-warmed recovery medium (RPMI supplemented with 10% FBS for 2D3 cells; AIM-V medium supplemented with 10% hAB for human primary T cells, and IMDM supplemented with 10% FBS for U266 cells) and rested for at least 20 min in a humidified 5% CO_2_ incubator at 37 °C. Before co-culture, cells were washed, resuspended in fresh medium and incubated for 4 h. When necessary, cells were electroporated without mRNA (mock) as a negative control.

### 4.4. Flow Cytometry

HLA-A*02:01 positivity of T2, U266, Raji-A2, K562-A2 cells was analyzed by direct staining using a PE-conjugated anti-human HLA-A*02 antibody (clone BB7-2; BioLegend, London, UK). HLA-A*02:01 expression on PBMC samples was detected by incubation with the supernatant of the hybridoma BB7.2 cell line (producer of anti-HLA-A*02 antibody, ATCC) for 15 min at room temperature. Then, cells were washed with FACS buffer (FACSFlow sheath fluid (BD Biosciences), 0.1% bovine serum albumin (BSA) (Sigma-Aldrich), 0.05% sodium azide (Merck, Overijse, Belgium), labeled with FITC-conjugated polyclonal rabbit anti-mouse immunoglobulins (Dako, Heverlee, Belgium) for 15 min at room temperature protected from light. WT1 expression was analyzed in samples from T2, U266, Raji-A2, and K562-A2 cell lines or electroporated U266 cells 24 h after *WT1* mRNA electroporation by intracellular staining. Cells were harvested for fixation and permeabilization using the Foxp3/Transcription factor staining buffer set (eBioscience, Life Technologies) according to the manufacturer’s instructions. Next, cells were labeled with unconjugated mouse anti-human WT1 monoclonal antibody (clone 6F-H2, Dako)—which recognizes an epitope within residues 1-181 of all isoforms of the full-length WT1 protein—followed by PE-conjugated polyclonal rabbit anti-mouse immunoglobulins (Dako). As a control, samples were only incubated with PE-conjugated polyclonal rabbit anti-mouse immunoglobulins. WT1-specific TCR surface expression was evaluated 24 h after *TCR* mRNA electroporation in 2D3 and primary CD8 T cells. For 2D3 cells, samples were labeled with FITC-conjugated anti-CD8 (BD Biosciences) and PE-conjugated anti-pan TCRαβ (Miltenyi Biotec) or isotype control mAb (BD Biosciences) for 15 min at room temperature. For primary CD8 T cells, samples were labeled with PE-conjugated WT1_37–45_ or WT1_126–134_ peptide/HLA-A*02:01 tetramers for 30 min at 37 °C [28]. Then, cells were washed and labeled with FITC-conjugated anti-CD8 and PerCP-conjugated anti-CD3 mAbs (BD Biosciences) for 15 min at room temperature. All samples were washed previous to analysis on a CytoFLEX cytometer (Beckman Coulter).

### 4.5. Peptide-Pulsing of Tumor Cells

Viable T2, U266, Raji-A2, and K562-A2 cells were harvested, washed once in serum-free IMDM medium, and resuspended using the same medium at a final concentration of 10^6^ cells/mL. Cells were split in tubes and pulsed with WT1_37–45_ (VLDFAPPGA) or WT1_126–134_ peptide (RMFPNAPYL) (JPT Peptide Technologies, Berlin, Germany) at decreasing concentrations of a ten-fold serial dilution from a concentration of 10 μg/mL for 60 min at room temperature under constant motion. After incubation, cells were washed and resuspended in IMDM supplemented with 10% FBS at a concentration of 5 × 10^5^ cells/mL.

### 4.6. Co-Cultures

Electroporated 2D3 or DSE primary CD8 T cells were co-cultured with peptide-pulsed T2, U266, Raji-A2 and K562-A2 cells or electroporated U266 cells in triplicate in 96-well round-bottom plates at an effector:target (E:T) ratio of 2:1 (2D3 cells) or 4:1 (primary CD8 T cells). 2D3 cells or primary CD8 T cells cultured alone served as negative controls. Co-cultures were incubated for 18–22 h at 37 °C in a humidified atmosphere supplemented with 5% CO_2_.

### 4.7. Analysis of Epitope-Specific T-Cell Activation

After co-culture, cells were harvested and analyzed for epitope-specific expression of the enhanced green fluorescent protein (EGFP; 2D3 cells) or expression of the activation markers CD137 and CD69 (primary CD8 T cells). Samples from 2D3 cell co-cultures were washed, incubated with PE-conjugated anti-CD8 for 15 min at room temperature. Then, samples were rewashed and stained with the nucleic acid dye 7-aminoactinomycin D (7-AAD; BD Biosciences) for 10 min at room temperature for the exclusion of nonviable cells before analysis on a CytoFLEX cytometer (Beckman Coulter). Samples from primary CD8 T cell co-cultures were washed and stained with anti-human PE-conjugated anti-CD137, PerCP-Cy5.5-conjugated anti-CD3, APC-Cy7-conjugated anti-CD69 (BD Biosciences) and Pacific Blue-conjugated anti-CD8 (Life Technologies) monoclonal antibodies and LIVE/DEAD fixable aqua dead cell stain kit (Thermo Fisher Scientific) for 15 min at room temperature. After incubation, cells were washed and analyzed using a FACSAria II cytometer (BD Biosciences).

### 4.8. Statistical Analysis

Data from flow cytometers were analyzed using FlowJo v10.2 software (TreeStar Inc, Ashland, OR, USA). Prism v5 software (GraphPad, San Diego, CA, USA) was used for graphing, statistical calculations and calculation of EC50 values. Data were analyzed using one-way analysis of variance (ANOVA) followed by Dunnett’s or Tukey’s post hoc test where applicable for multiple comparisons. Results were considered to be statistically significant when *p*-value was less than 0.05. * indicates *p* < 0.05, ** indicates *p* < 0.01, *** indicates *p* < 0.001 and **** indicates *p* < 0.0001.

## 5. Conclusions

Our study demonstrates the relevance of comparing the APCs used in T-cell assays and the influence they may have when evaluating T-cell functional avidity. Here, we provide a versatile model to evaluate HLA-A2-restricted WT1 epitope-specific responses by TCR-engineered T cells based on the combination of a tumor cell-based APC with a rapid engineering method such as mRNA electroporation. This model could be valuable for the screening and selection of WT1-specific high-avidity TCRs intended for TCR-engineered therapies without the need for primary APCs. It can potentially be used to analyze other TAA-specific T cells, in particular, for those T cells with low circulating levels that are reactive against tumor-associated autoantigens in the style of WT1. Eventually, this platform could provide the basis for the development of an immunomonitoring tool to evaluate TAA-specific T-cell activity in clinical trials using TAA mRNA-electroporated DC vaccines for cancer immunotherapy.

## Supplementary Materials

The following are available online at https://www.mdpi.com/2072-6694/12/2/256/s1, Figure S1: WT1-specific TCR expression on 2D3 and primary CD8 T cells.
