# Supplementary material for: Rapid Assessment of Functional Avidity of Tumor-Specific T Cell Receptors Using an Antigen-Presenting Tumor Cell Line Electroporated with Full-Length Tumor Antigen mRNA
Source: Cancers (Basel). 2020 Jan 21;12(2):256. doi: 10.3390/cancers12020256 (PMC7072428; doi:10.3390/cancers12020256)
Supplement: Supplementary file 1 [file cancers-12-00256-s001.pdf]

Supplementary Materials

Rapid Assessment of Functional Avidity of Tumor-Specific T Cell Receptors using an Antigen-Presenting Tumor Cell Line Electroporated with Full-Length Tumor Antigen mRNA

Diana Campillo-Davo, Maarten Versteven, Gils Roex, Hans De Reu, Sanne van der Heijden, Sébastien Anguille, Zwi N. Berneman, Viggo F.I. Van Tendeloo and Eva Lion

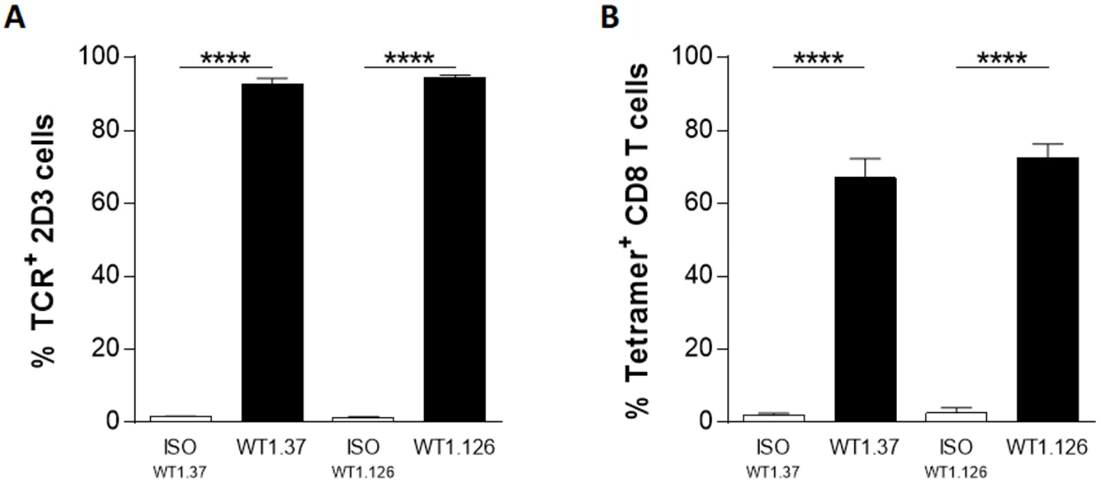

Figure S1. WT1-specific TCR expression on 2D3 and primary CD8 T cells. Surface expression of WT1-specific TCR in 2D3 cells (**A**) or primary CD8 T cells (**B**) was analyzed 24 h after electroporation with either WT137-45- (WT1.37) or WT1126-134-specific (WT1.126) codon-optimized TCR mRNA by anti-human TCR $\alpha\beta$ antibody staining (A) or by HLA:A*02:01/WT137-45 (WT1.37) or HLA:A*02:01/WT1126-134 (WT1.126) tetramer staining (B). Mean ± SEM of 10 independent replicates (A) and of 8 donors (B) is shown. WT1, Wilms' tumor 1; ISO, isotype. ****, $p < 0.0001$.

© 2020 by the authors. Licensee MDPI, Basel, Switzerland. This article is an open access article distributed under the terms and conditions of the Creative Commons Attribution (CC BY) license (http://creativecommons.org/licenses/by/4.0/).
